# Preoperative serum low-density lipoprotein cholesterol is an independent prognostic factor in patients with renal cell carcinoma after nephrectomy

**DOI:** 10.1186/s12944-023-01791-6

**Published:** 2023-02-22

**Authors:** Fan Cui, Huiyu Zhou, Dingyang Lv, Jie Wen, Qian Gong, Yi Rong, Yinbo Kang, Mohan Jia, Weibing Shuang

**Affiliations:** 1 First Clinical Medical College, Shanxi Medical University, Taiyuan, China; 2 Department of Urology, The First Hospital of Shanxi Medical University, No. 85, JieFang South Road, Yingze District, Taiyuan, 030001 China

**Keywords:** Renal cell carcinoma, Low-density lipoprotein cholesterol, Nephrectomy, Prognosis

## Abstract

**Background:**

Little is known about the association between the preoperative low-density lipoprotein cholesterol (LDL-C) level and prognosis in patients with renal cell carcinoma (RCC) after nephrectomy, and its prognostic value needs to be elucidated.

**Methods:**

The clinical and follow-up data of 737 RCC patients who underwent nephrectomy were retrospectively analyzed. The optimal cut-off LDL-C level was determined using X-tile, and then patients were divided into low and high LDL-C groups. The association between LDL-C levels and survival of RCC patients was assessed using the Kaplan-Meier method and Cox regression analysis.

**Results:**

The optimal cut-off LDL-C level was 1.93 mmol/L, and patients were divided into the low (≤ 1.93 mmol/L) and high LDL-C (> 1.93 mmol/L) groups. The Kaplan-Meier analysis showed that patients in the low LDL-C group had significantly shorter overall survival (OS), cancer-specific survival (CSS) and recurrence-free survival (RFS) than those in the high LDL-C group (*P* = 0.001, *P* = 0.001, and *P* = 0.003, respectively). The COX univariate analysis showed that the preoperative LDL-C level was closely associated with OS, CSS, and RFS in RCC patients (*P* = 0.002, *P* = 0.003, and *P* = 0.005, respectively). The multivariate analysis showed that the preoperative LDL-C level was an independent factor for predicting survival (OS, CSS and RFS) in RCC patients after nephrectomy. The low preoperative LDL-C levels predicted worse OS (hazard ratio [HR]: 2.337; 95% confidence interval [CI]: 1.192–4.581; *P* = 0.013), CSS (HR: 3.347; 95% CI: 1.515–7.392; *P* = 0.003), and RFS (HR: 2.207; 95% CI: 1.178–4.132; *P* = 0.013).

**Conclusions:**

The preoperative LDL-C level is an independent factor for the prognosis of RCC patients after nephrectomy, and low preoperative LDL-C levels predict worse survival (OS, CSS, and RFS).

## Introduction

Renal cell carcinoma (RCC) is the most common solid tumor of the kidney, accounting for approximately 85% of all renal tumors and 95% of all renal malignancies [1–3]. The incidence of kidney cancer has increased worldwide in the past 30 years [4]. In 2020, there were approximately 431,288 new cases of kidney cancer in the world, accounting for 2.2% of all new cases of cancer, and there were approximately 179,318 deaths due to kidney cancer, accounting for 1.8% of all deaths due to cancer [5]. Kidney cancer has become a great global burden of disease [4].

Nephrectomy is the major treatment option for localized renal cancer. However, nearly 30% of patients experience tumor recurrence or metastasis following nephrectomy, leading to treatment failure and shortened survival of patients [6, 7]. At present, tumor grade, TNM stage, and Karnofsky scores are common prognostic factors for the prognosis of RCC patients. Based on these factors, prognostic models have been developed to stratify the risk of RCC patients. However, the prognostic ability of these models is relatively low [8, 9]. It is expected that prognostic models can be improved by integrating more routine laboratory parameters, such as platelet volume, platelet count, neutrophil count, and serum albumin [8–10].

Lipid metabolism plays an important role in cancer [11, 12]. Changes in blood lipid levels are associated with the risk, pathological features, and prognosis of various cancers, such as colon cancer [13], gastric cancer [14], prostate cancer [15], and breast cancer [16]. Low-density lipoprotein cholesterol (LDL-C) is a routine laboratory parameter of lipid metabolism in clinical practice, and it is highly associated with the prognosis of a variety of cancers, such as lung cancer [17], esophageal cancer [18], ovarian cancer [19], breast cancer [20], and colon cancer [21]. Moreover, LDL-C can promote the proliferation and migration of cancer cells [22, 23]. Recently, high-density lipoprotein cholesterol (HDL-C) and total cholesterol (TC) have been considered independent factors for the prognosis of RCC patients [24, 25]. However, little is known about the prognostic value of LDL-C in RCC patients.

## Materials and methods

### Study population

**Fig. 1:**
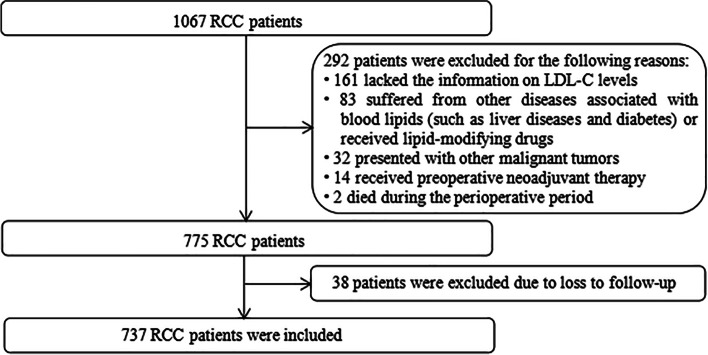
The process of patient selection for the retrospective study. Abbreviations: RCC renal cell carcinoma, LDL-C low-density lipoprotein cholesterol

From 2013 to 2021, there were 1067 patients diagnosed with RCC who underwent nephrectomy at the Department of Urology, of the First Hospital of Shanxi Medical University, and their clinical data were collected and analyzed. A total of 330 patients were excluded based on the following criteria, including (1) incomplete clinical data (*n* = 161), (2) history of diseases associated with blood lipids (such as liver diseases and diabetes) or use of lipid-modifying drugs (*n* = 83), (3) presentation with other malignant tumors (*n* = 32), (4) receiving preoperative neoadjuvant therapy (*n* = 14), (5) death during the perioperative period (*n* = 2), and (6) loss to follow-up (*n* = 38). Finally, a total of 737 patients were included in this study (Fig. 1). This study was approved by the Ethics Committee of the First Hospital of Shanxi Medical University, and written informed consent was obtained from all patients.

### Data collection

The clinical characteristics collected in these patients included gender, body mass index (BMI), age, history of hypertension, smoking history, tumor size, tumor subtype, tumor laterality, Fuhrman grade, T stage, N stage, type of surgery, and preoperative LDL-C levels. Patients who have smoked more than 100 cigarettes during their lifetime were considered to have a smoking history [26]. The tumor subtype was determined based on the 2016 World Health Organization classification of urogenital tumors [27]. T and N stages were determined based on the guidelines established by the American Joint Committee on Cancer [28]. Fuhrman grades were determined by the Fuhrman grading system [29]. Preoperative LDL-C levels were measured 1 day after hospital admission of patients. The optimal cut-off value for the preoperative LDL-C levels was determined using X-tile 3.6.1 (Yale University, USA) [30], which was used to classify RCC patients into low and high LDL-C groups.

### Follow-up

Patients were followed up by telephone and outpatient review, which included physical examination, laboratory tests, and imaging. Follow-up was performed every 3 months in the first 3 years after hospital discharge, every 6 months during years 4 through 5, and then annually until death or September 30, 2022. Overall survival (OS) was calculated from the date of surgery to the date of the last follow-up or death. Cancer-specific survival (CSS) was calculated from the date of surgery to the date of the last follow-up or death due to cancer. Recurrences included local recurrence and distant metastasis after nephrectomy. Recurrence-free survival (RFS) was calculated from the date of surgery to the date of recurrence or the last follow-up.

### Statistical analysis

Continuous variables were presented as mean ± standard deviation (SD) and the difference between the two groups was evaluated by Student’s t-test. Categorical variables were presented as numbers (percentages) and the difference between the two groups was evaluated by the Chi-squared test. Survival times (OS, CSS, and RFS) of patients were estimated using the Kaplan-Meier method, and survival between groups was compared using the log-rank test. Postoperative prognostic factors in RCC patients were analyzed using the COX univariate and multivariate analyses. Differences were considered statistically significant when *P* < 0.01 for COX univariate analysis and *P* < 0.05 for the rest of the statistical analyses. Statistical analyses were performed using SPSS 26.0 (IBM Corporation, Armonk, New York, USA).

## Results

### The characteristics of patients

Among the 737 patients, 476 (64.6%) and 261 (35.4%) were male and female, respectively. The mean age of all patients was 57.7 years (range 27–86 years). The optimal cut-off value for preoperative LDL-C levels was 1.93 mmol/L, and patients were classified into low (≤ 1.93 mmol/L) and high LDL-C (> 1.93 mmol/L) groups, with 94 and 643 patients in the two groups, respectively. The median follow-up was 38 months, ranging from 1 to 112 months. During follow-up, 48 (6.5%) patients died, of which 31 (4.2%) died of cancer and 54 (7.3%) relapsed.

### Comparison of patient characteristics between low and high LDL-C groups

There were significant differences in gender, T stage, age, and BMI between the two groups (*P* < 0.05). The mean age of patients was older in the low LDL-C group than in the high LDL-C group, and the mean BMI of patients was lower in the low LDL-C group than in the high LDL-C group. There were no significant differences in smoking history, history of hypertension, tumor laterality, tumor subtype, N stage, Fuhrman grade, and tumor size between the two groups (*P* > 0.05) (Table 1).

**Table 1:** Comparison of the characteristics of RCC patients between the low and high LDL-C groups

Variables	All patients (*n* = 737)	LDL-C ≤ 1.93 (*n* = 94)	LDL-C > 1.93 (*n* = 643)	*P*
	n (%)			
Gender				0.002^*^
Male	476 (64.6)	74 (78.7)	402 (62.5)	
Female	261 (35.4)	20 (21.3)	241 (37.5)	
Smoking history				0.100
Yes	199 (27.0)	32 (34.0)	167 (26.0)	
No	538 (73.0)	62 (66.0)	476 (74.0)	
Hypertension				0.208
Yes	301 (40.8)	44 (46.8)	257 (40.0)	
No	436 (59.2)	50 (53.2)	386 (60.0)	
Laterality				0.914
Left	349 (47.4)	45 (47.9)	304 (47.3)	
Right	388 (52.6)	49 (52.1)	339 (52.7)	
Tumor subtype				0.548
Clear	678 (92.0)	85 (90.4)	593 (92.2)	
Non-clear	59 (8.0)	9 (9.6)	50 (7.8)	
T stage				0.031^*^
T1	641 (87.0)	75 (79.8)	566 (88.0)	
T2	71 (9.6)	12 (12.8)	59 (9.2)	
T3-T4	25 (3.4)	7 (7.4)	18 (2.8)	
N stage				1.000
N0/Nx	729 (98.9)	93 (98.9)	636 (98.9)	
N+	8 (1.1)	1 (1.1)	7 (1.1)	
Fuhrman grade				0.207
1	127 (17.2)	10 (10.6)	117 (18.2)	
2	436 (59.2)	56 (59.6)	380 (59.1)	
3	152 (20.6)	24 (25.5)	128 (19.9)	
4	22 (3.0)	4 (4.3)	18 (2.8)	
Type of surgery				0.151
RN	493 (66.9)	69 (73.4)	424 (65.9)	
PN	244 (33.1)	25 (26.4)	219 (34.1)	
	mean ± SD			
Age	57.7 ± 10.8	60.2 ± 11.7	57.4 ± 10.7	0.017^*^
BMI	25.0 ± 3.4	24.4 ± 3.1	25.1 ± 3.5	0.045^*^
Tumor size	4.4 ± 2.3	4.7 ± 2.4	4.3 ± 2.3	0.120

Continuous variables were analyzed by the Student’s t-test, while categorical variables were analyzed by the Chi-squared test

Abbreviations: *LDL-C* Low-density lipoprotein cholesterol, *RN* Radical nephrectomy, *PN* Partial nephrectomy, *SD* Standard deviation, *BMI* Body mass index

^*^ indicates *P* < 0.05

### Prognostic value of the preoperative LDL-C level in RCC patients after nephrectomy

During follow-up, 14 (14.9%) patients died in the low LDL-C group, of which 10 (10.6%) died of cancer. In contrast, 34 (5.3%) patients died in the high LDL-C group, of which 21 (3.3%) died of cancer. In addition, 14 (14.9%) patients experienced RCC recurrence in the low LDL-C group, while 40 (6.2%) patients experienced RCC recurrence in the high LDL-C group.

**Fig. 2:**
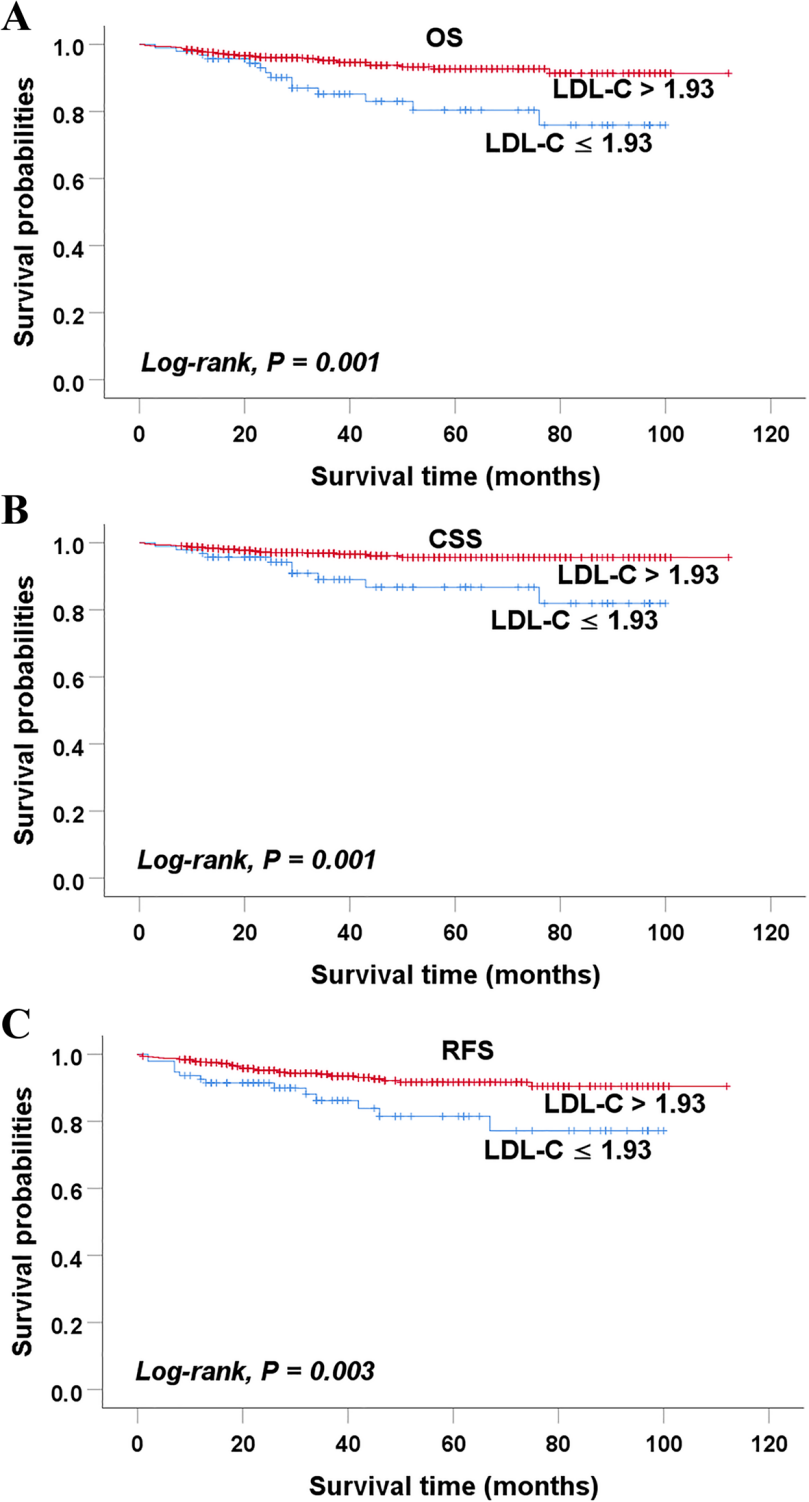
Kaplan-Meier curves of survivals of patients in the low-LDL-C and high -LDL-C groups (**A**) OS (**B**) CSS (**C**) RFS. Abbreviations: LDL-C low-density lipoprotein cholesterol, OS overall survival, CSS cancer-specific survival, RFS recurrence-free survival. There were significant differences in OS, CSS, and RFS between two groups (*P* < 0.05)

As for OS, the Kaplan-Meier analysis showed that the 5-year survival rate (83.0%) of the low LDL-C group was significantly worse than that (92.7%) of the high LDL-C group (*P* = 0.001) (Fig. 2A). The univariate analysis showed that age, smoking history, tumor size, tumor subtype, T stage, N stage, Fuhrman grade and preoperative LDL-C level were significantly associated with OS (all *P* < 0.01). Furthermore, the multivariate analysis showed that age, tumor size, tumor subtype, smoking history, N stage, Fuhrman grade, and preoperative LDL-C were independent prognostic factors for OS. Low preoperative LDL-C levels predicted worse OS (hazard ratio [HR]: 2.337; 95% confidence interval [CI]: 1.192–4.581; *P* = 0.013) (Table 2).

**Table 2:** Univariate and multivariate COX analysis of OS in RCC patients after nephrectomy

Variables	Univariate analysis		Multivariate analysis	
	HR (95% CI)	*P*	HR (95% CI)	*P*
Gender (male vs female)	2.158 (1.075–4.331)	0.031^*^		
Age	1.041 (1.012–1.072)	0.006^**^	1.053 (1.020–1.087)	0.001^**^
BMI	0.950 (0.886–1.044)	0.288		
Smoking history (no vs yes)	0.297 (0.168–0.524)	< 0.001^**^	0.287 (0.153–0.535)	< 0.001^**^
Hypertension (no vs yes)	1.010 (0.566–1.801)	0.974		
Laterality (left vs right)	1.140 (0.647–2.008)	0.650		
Tumor size	1.393 (1.284–1.511)	< 0.001^**^	1.515 (1.289–1.782)	< 0.001^**^
Tumor subtype (non-clear vs clear)	2.959 (1.432–6.112)	0.003^**^	4.215 (1.894–9.377)	< 0.001^**^
T stage		< 0.001^**^		0.316
T1	Reference		Reference	
T2	5.093 (2.628–9.870)	< 0.001^**^	0.455 (0.152–1.363)	0.159
T3-T4	6.815 (2.974–15.616)	< 0.001^**^	0.463 (0.146–1.471)	0.192
N stage (N0/Nx vs N+)	0.113 (0.035–0.365)	< 0.001^**^	0.104 (0.028–0.379)	0.001^**^
Fuhrman grade		< 0.001^**^		0.011^*^
1	Reference		Reference	
2	1.716 (0.582–5.066)	0.328	1.137 (0.372–3.477)	0.821
3	5.827 (1.967–17.258)	0.001^**^	2.370 (0.774–7.259)	0.131
4	13.433 (3.757–48.021)	< 0.001^**^	5.321 (1.407–20.127)	0.014^*^
Type of surgery (PN vs RN)	0.901 (0.299–2.720)	0.853		
LDL-C (≤ 1.93 vs > 1.93)	2.740 (1.469–5.110)	0.002^**^	2.337 (1.192–4.581)	0.013^*^

Abbreviations: *OS* Overall survival, *HR* Hazard ratio, *CI* Confidence interval, *BMI* Body mass index, *PN* Partial nephrectomy, *RN* Radical nephrectomy, *LDL-C* Low-density lipoprotein cholesterol

^* *^ indicates *P* < 0.01, ^*^ indicates *P* < 0.05

As for CSS, the Kaplan-Meier analysis showed that the 5-year survival rate (86.7%) of the low LDL-C group was significantly worse than that (95.6%) of the high LDL-C group (*P* = 0.001) (Fig. 2B). The univariate analysis showed that tumor size, tumor subtype, T stage, N stage, Fuhrman grade, and preoperative LDL-C level were significantly associated with CSS (all *P* < 0.01). Furthermore, the multivariate analysis indicated that tumor size, tumor subtype, N stage, and preoperative LDL-C level were independent prognostic factors for CSS. Low LDL-C levels were closely related to worse CSS (HR: 3.347; 95% CI: 1.515–7.392; *P* = 0.003) (Table 3).

**Table 3:** Univariate and multivariate COX analysis of CSS in RCC patients after nephrectomy

Variables	Univariate analysis		Multivariate analysis	
	HR (95% CI)	*P*	HR (95% CI)	*P*
Gender (male vs female)	1.382 (0.636–3.002)	0.414		
Age	1.042 (1.005–1.080)	0.025^*^		
BMI	0.937 (0.833–1.055)	0.284		
Smoking history (no vs yes)	0.427 (0.210–0.867)	0.019^*^		
Hypertension (no vs yes)	1.212 (0.581–2.530)	0.609		
Laterality (left vs right)	0.935 (0.461–1.898)	0.853		
Tumor size	1.391 (1.259–1.538)	< 0.001^**^	1.345 (1.133–1.597)	0.001^**^
Tumor subtype (non-clear vs clear)	3.719 (1.601–8.637)	0.002^**^	3.199 (1.252–8.171)	0.015^*^
T stage		< 0.001^*^		0.725
T1	Reference		Reference	
T2	4.689 (2.033–10.813)	< 0.001^**^	0.578 (0.151–2.217)	0.424
T3-T4	7.450 (2.763–20.089)	< 0.001^**^	0.690 (0.153–3.102)	0.628
N stage (N0/Nx vs N+)	0.116 (0.028–0.488)	0.003^**^	0.204 (0.044–0.957)	0.044^*^
Fuhrman grade		< 0.001^**^		0.051
1	Reference		Reference	
2	4.855 (0.636–37.031)	0.128	3.415 (0.440–26.522)	0.240
3	13.886 (1.795–107.395)	0.012^*^	6.677 (0.833–53.492)	0.074
4	33.375 (3.705–300.621)	0.002^**^	13.476 (1.434–126.634)	0.023^*^
Type of surgery (PN vs RN)	0.344 (0.065–1.822)	0.210		
LDL-C (≤ 1.93 vs > 1.93)	3.197 (1.504–6.794)	0.003^**^	3.347 (1.515–7.392)	0.003^**^

Abbreviations: *CSS* Cancer-specific survival, *HR* Hazard ratio, *CI* Confidence interval, *BMI* Body mass index, *PN* Partial nephrectomy, *RN* Radical nephrectomy, *LDL-C* Low-density lipoprotein cholesterol

^* *^ indicates *P* < 0.01, ^*^ indicates *P* < 0.05

Regarding RFS, the Kaplan-Meier analysis showed that the 5-year survival rate (81.5%) of the low LDL-C group was significantly worse than that (91.6%) of the high LDL-C group (*P* = 0.003) (Fig. 2C). The univariate analysis indicated that tumor size, T stage, N stage, Fuhrman grade, and preoperative LDL-C level were highly associated with RFS (all *P* < 0.01). The multivariate analysis revealed that tumor size, N stage, Fuhrman grade, and preoperative LDL-C level were independent prognostic factors for RFS. Low preoperative LDL-C levels were strongly associated with worse RFS (HR: 2.207; 95% CI: 1.178–4.132; *P* = 0.013) (Table 4).

**Table 4:** Univariate and multivariate COX analysis of RFS in RCC patients after nephrectomy

Variables	Univariate analysis		Multivariate analysis	
	HR (95% CI)	*P*	HR (95% CI)	*P*
Gender (male vs female)	1.349 (0.752–2.420)	0.315		
Age	1.018 (0.992–1.044)	0.180		
BMI	0.917 (0.837–1.005)	0.064		
Smoking history (no vs yes)	0.498 (0.289–0.857)	0.012^*^		
Hypertension (no vs yes)	1.054 (0.610–1.822)	0.850		
Laterality (left vs right)	0.844 (0.492–1.448)	0.538		
Tumor size	1.341 (1.237–1.455)	< 0.001^**^	1.393 (1.202–1.614)	< 0.001^**^
Tumor subtype (non-clear vs clear)	2.197 (1.037–4.657)	0.040^*^		
T stage		< 0.001^**^		0.267
T1	Reference		Reference	
T2	4.059 (2.142–7.691)	< 0.001^**^	0.389 (0.124–1.223)	0.106
T3-T4	4.686 (1.970–11.146)	< 0.001^**^	0.602 (0.187–1.931)	0.393
N stage (N0/Nx vs N+)	0.081 (0.029–0.227)	< 0.001^**^	0.130 (0.041–0.413)	0.001^**^
Fuhrman grade		< 0.001^**^		0.011^*^
1	Reference		Reference	
2	1.878 (0.721–4.892)	0.197	1.548 (0.586–4.093)	0.378
3	3.498 (1.265–9.677)	0.016^*^	1.913 (0.666–5.491)	0.228
4	12.868 (4.051–40.873)	< 0.001^**^	6.247 (1.874–20.826)	0.003^**^
Type of surgery (PN vs RN)	0.853 (0.346–2.100)	0.729		
LDL-C (≤ 1.93 vs > 1.93)	2.412 (1.312–4.435)	0.005^**^	2.207 (1.178–4.132)	0.013^*^

Abbreviations: *RFS* Recurrence-free survival, *HR* Hazard ratio, *CI* Confidence interval, *BMI* Body mass index, *PN* Partial nephrectomy, *RN* Radical nephrectomy, *LDL-C* Low-density lipoprotein cholesterol

^* *^ indicates *P* < 0.01, ^*^ indicates *P* < 0.05

## Discussion

Metabolic reprogramming in cancer cells is important for their rapid growth and proliferation [31]. As for lipid metabolic reprogramming, cancer cells increase lipid uptake, biosynthesis, and storage, but decrease lipid catabolism and efflux [11]. Lipid metabolism disorders and lipid metabolic reprogramming are closely associated with the invasion and metastasis of RCC [7].

This study showed that patients with low LDL-C levels had worse survival (OS, CSS, and RFS). The phenomenon might be related to lipid metabolism reprogramming in RCC patients. Cholesterol is a major structural component of cell membranes, and it is also a key substance in cellular energy metabolism for growth [31, 32]. Cholesterol can be synthesized by cells or internalized via low-density lipoprotein (LDLs) [11]. After binding to low-density lipoprotein receptors (LDLRs) on the cell membrane, LDLs enters the cells and is hydrolyzed by lysosomes to release free cholesterols [20, 33]. Normal cells can control de novo cholesterol synthesis and uptake of extracellular LDLs to maintain intracellular cholesterol homeostasis [32]. However, cancer cells can increase cholesterol synthesis and LDLR expression, which increase intracellular cholesterol levels but decrease serum TC and LDL-C levels [32, 33]. Recent studies have found that low preoperative serum TC levels are highly associated with tumor aggressiveness and poor prognosis [7, 24]. In addition, LDLRs have also been considered an independent prognostic factor for cancer patients [7, 32, 33].

Lipid accumulation is one of the hallmarks of clear cell renal cell carcinoma (ccRCC), a common subtype of RCC [31]. LDLR and LDLR-related proteins are overexpressed in ccRCC patients [34, 35]. These studies indirectly indicate a possible association between RCC progression and LDL uptake.

It is reported that anatomical and histological factors are associated with the prognosis of RCC patients [8]. Here, the results confirmed that tumor size and N stage were prognostic factors for OS, CSS, and RFS in RCC patients. Large tumor size and lymph node metastasis predicted a worse prognosis.

The previous study has explored the prognostic value of serum lipid-profile in RCC, yet has not found that LDL-C is associated with the prognosis in patients with RCC [36]. This study showed that low preoperative LDL-C levels predicted a poor prognosis in RCC patients after nephrectomy, suggesting that lipid levels should be continuously monitored. Similarly, there is a strong correlation between low preoperative TC levels and the survival of RCC patients after nephrectomy [24]. Because cholesterol metabolism is closely related to LDL, lipoprotein therapy might be a promising treatment for RCC patients [11, 17]. Some inhibitors targeting lipid metabolism in RCC such as SR9243 and liver X receptor-623 have been confirmed to be effective in vivo or in vitro trials, but no drugs targeting LDL or LDLR have been reported [7].

### Comparisons with other studies and what does the current work add to the existing knowledge

Serum TC and HDL-C levels have been reported to be associated with the prognosis in RCC patients [24, 25]. This is the first study that demonstrated the association between preoperative LDL-C levels and the prognosis of RCC patients after nephrectomy. The results can not only provide evidence for establishing a prognostic model for RCC patients based on lipid levels but also help RCC patients with low LDL-C levels receive more effective postoperative care after nephrectomy.

### Strengths and limitations

This study innovatively assessed the possible association between preoperative serum LDL-C levels and the prognosis of RCC patients after nephrectssomy. The results could help clinicians accurately assess the prognosis of RCC patients and provide evidence for lipid monitoring and lipoprotein therapy in RCC patients with low preoperative LDL-C levels. This study had some limitations. First, this is a retrospective study, which may have a patient selection bias. Second, this is a single-center study with Asians. It is expected that multicenter studies involved with large samples and other race/ethnic groups can validate the relevant findings.

## Conclusions

This study demonstrated that preoperative serum LDL-C levels could be used as an independent predictor for the prognosis of RCC patients. Low preoperative LDL-C levels predicted a worse prognosis of RCC patients after nephrectomy. LDL-C levels can be included in the prognostic models to improve their predictability for the prognosis of RCC patients. And careful attention should be paid to blood lipid indices in RCC patients with low preoperative LDL-C levels. Lipoprotein therapy is expected to be a promising treatment for dyslipidemic RCC patients.

## Data Availability

The data used in the current study are available from the corresponding author on reasonable request.

## Abbreviations

LDL-C: Low-density lipoprotein cholesterol
RCC: Renal cell carcinoma
OS: Overall survival
CSS: Cancer-specific survival
RFS: Recurrence-free survival
HR: Hazard ratio
CI: Confidence interval
HDL-C: High-density lipoprotein cholesterol
TC: Total cholesterol
BMI: Body mass index
SD: Standard deviation
RN: Radical nephrectomy
PN: Partial nephrectomy
LDL: Low-density lipoprotein
LDLR: Low-density lipoprotein receptor
ccRCC: Clear cell renal cell carcinoma
